# Dialysis Monitoring of Ionic Strength and Denaturant Effects, and Their Reversibility, for Various Classes of Macromolecules

**DOI:** 10.1021/acs.biomac.4c00583

**Published:** 2024-07-29

**Authors:** Curtis W. Jarand, Melanie J. McLeod, Wayne F. Reed

**Affiliations:** Tulane University, New Orleans, Louisiana 70115, United States

## Abstract

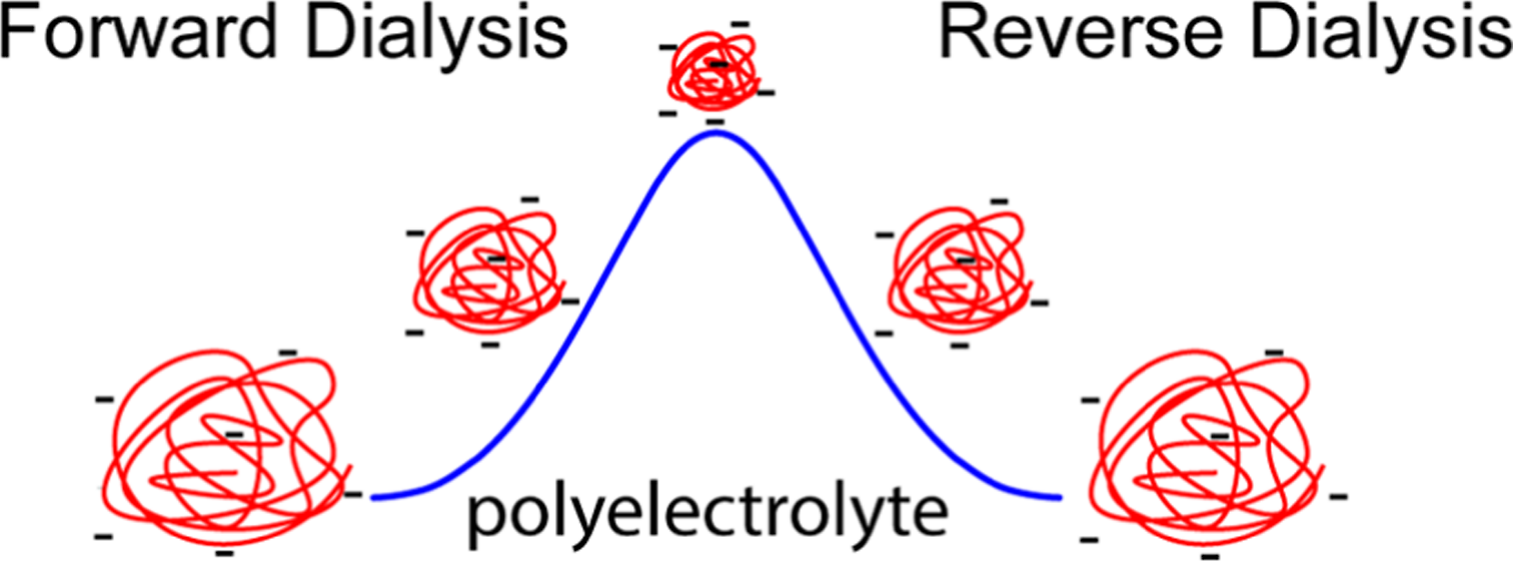

Monitoring membrane-mediated dialysis in real time with static and dynamic light scattering revealed distinctive differences, including reversibility/irreversibility, in the effects of ionic strength (NaCl) and the denaturant guanidine-HCl (Gd) on a synthetic polyelectrolyte and several types of biomacromolecules: protein, polysaccharide, and polyampholyte. Dialysis cycles against aqueous NaCl and Gd, and reverse back to the original aqueous solution, were monitored. The behavior of Na-polystyrenesulfonate was reversible and yielded a detailed polymer physics description. The biomacromolecules additionally showed hydrogen-bonding/hydrophobic (HP) interactions. An interpretive model was developed that considers the interplay among polyelectrolyte, polyampholyte, and HP potential energies in determining the different associative, aggregative, and dissociative behaviors. NaCl isolated purely electrostatic effects, whereas Gd combined electrostatic and HP effects. Some macromolecules showed partially reversible behavior, and others were completely irreversible. The dialysis monitoring method should prove useful for investigating fundamental macromolecular and colloid properties and for drug formulation and stability optimization.

## 1 Introduction

Charged macromolecules, polyelectrolytes, are a field of continuing intensive study, both for their fundamental interest, and for many practical applications in medicine, polymeric materials, batteries, food and agriculture, and much more.^1−4^ Polyelectrolytes respond to the added electrolytes and denaturants in different ways. Polyelectrolyte coils with charges of a single sign shrink as ionic strength increases and charges are shielded, whereas polyampholytes, having charges of both signs, generally increase in coil size as ionic strength increases and shielding decreases attractive forces between oppositely charged groups. Certain biomacromolecules, proteins, polysaccharides, RNA, and DNA—can undergo changes in size and also reversibly or irreversibly aggregate when ionic strength increases. For molecules with secondary structure, a denaturant, such as guanidine hydrochloride (Gd), can cause partial or complete loss of secondary structure and, in some cases, aggregation. Similarly, the tertiary structures of some proteins can be affected by denaturants.

An important question surrounding these changes in characteristics as ionic strength and denaturant vary is whether the changes are reversible, partially reversible, or irreversible. While it is simple to increase the concentration of solutes, such as electrolytes and denaturants, and to measure corresponding changes in the characteristics of macromolecules and colloids, removing these agents requires a method, such as membrane dialysis or ultrafiltration. The current work uses a device that enables macromolecular characteristics to be spectroscopically monitored in real time during dialysis or other membrane-mediated processes. Hence, it is possible to continuously monitor the addition to a macromolecular or colloidal solution of an agent, such as an electrolyte or denaturant, and then to subsequently monitor the reverse process. The cycle can also start with the agent already present in the macromolecular solution, dialyzing it away against a simple aqueous solution and then reversing the process and dialyzing the agent back into the solution. The spectroscopic detection means used can be static and dynamic light scattering (SLS and DLS, respectively), fluorescence, UV/visible absorption, circular dichroism, and any other instrument that can accept a 1 cm cuvette.

The main objectives of this work are (i) to introduce spectroscopic monitoring during membrane dialysis, (ii) to apply it to a variety of biomacromolecules, as well as one synthetic macromolecule, to monitor and quantify contrasting behavior among these during dialysis, using NaCl as a simple electrolyte and guanidine-HCl (Gd) as a chaotropic agent, and (iii) to seek a dimensionless interpretive model framework for understanding the results. The model here is restricted to intermolecular interactions. Further work can go into extending the model to intramolecular interactions, building an absolute energetic model, and applying the monitoring method in greater detail to specific macromolecules together with complementary techniques.

The macromolecules chosen to explore this monitored dialysis method are the following.

1) A simple synthetic polyelectrolyte. The basic solution properties of polystyrenesulfonate (PSS) as a function of continuously changing ionic strength are measured and interpreted in terms of well-known polymer physics approaches. This provides the simplest baseline system for assessing the dialysis approach.
2) A natural polyelectrolyte. The polysaccharide alginate was chosen and is expected to show polyelectrolyte properties similar to the synthetic homopolyelectrolyte PSS but also more complex behavior due to possible secondary structure and ability to self-associate, despite being a highly charged polyanion.
3) A natural polyampholyte. The behavior of this (gelatin was chosen) contrasts with that of a simple polyelectrolyte because of (i) its polyampholyte nature and (ii) secondary structure.
4) A selection of proteins was subjected to monitored dialysis to observe fundamental differences in their behavior toward a simple electrolyte (NaCl) and a chaotropic agent (Gd).

Proteins denature under certain conditions, including high temperature, extreme pH, mechanical stress, and in solution with compounds such as guanidine hydrochloride and urea, leading to loss of their globular native state.^5−7^ Protein aggregation due to denaturation is a major problem in the discovery and development of biologic drugs, and the method presented here may aid in identifying which formulation conditions provide optimum protein stability.^8,9^ A number of mechanistic models for protein aggregation exist,^10,11^ and solution conditions, including ionic strength, pH, and the presence of surfactants and other excipients must be found in order to arrive at stable therapeutic formulations.^12−16^

(i) Bovine serum albumen (BSA) was chosen because it is easily obtained and widely used for a variety of research purposes. Serum albumen proteins transport fatty acids and other molecules as well as act as antioxidants and anticoagulants.^17^
(ii) Lysozyme is a component of the innate immune system and is an antimicrobial enzyme.
(iii) Immunoglobulin (IgG) is an antibody that aids in controlling infection, neutralizing toxins, and more. Each individual IgG contains two identical antigen-binding sites, and there are four subclasses of IgG (IgG1, IgG2, IgG3, and IgG4) that have different (and sometimes opposing) properties. It was surmised that IgG may have more complex behavior under dialysis with NaCl and Gd than the other proteins.
iv) Proteinase K enzymatically cleaves peptide bonds adjacent to the carboxyl group of aliphatic and aromatic amino acids.^18^ It is used to inactivate endonuclease enzymes during DNA purification as well as to digest brain tissue for detection of proteinase-resistant prion proteins.^19^
v) Caseins are a family of phosphoproteins with net negative charge in the solutions in this work. It does not have secondary structure, nor any cysteine bonds, so that there is no tertiary structure, and it is not a globular protein. It is a natural emulsifier in milk^20^ and self-assembles into micelles.^21^ The micelles formed from low molar mass κ-casein molecules are spherical.

## 2 Materials and Methods

### 2.1 Macromolecules

PSS was synthesized by Julia S. Siqueira using KPS-initiated free-radical polymerization.

High viscosity sodium alginate was obtained from Alfa Aesar/Thermo Fisher Scientific (J61887, Lot M30G001). Its molar mass is on the order of 10^6^ g/mol.

Gelatin was supplied by Sanofi Bioindustries (Baupte, Normandy, France, now part of Cargill, Inc.), with a molar mass of approximately 150,000 g/mol. It was dissolved at 1 mg/mL in 10 mM NaCl concentrations. Gelatin is a fibrous protein, so it requires heating to dissolve. Solutions were placed in an orbital shaker at 40 °C for 2 h and then filtered with a 5 μm syringe filter immediately after cooling.

BSA was from Sigma-Aldrich in two preparations; one had a purity reported as over 99%, purified by gel electrophoresis, and the other a purity of 98%. BSA was dissolved at 2 mg/mL in phosphate buffer PBS (NaH_2_PO_4_–Na_2_HPO_4_) or Tris/Trizma buffer systems. BSA will dissolve at any pH between 5 and 9 at concentrations of below 30 mg/mL.

Hen egg white lysozyme was purchased from ThermoFisher Scientific. The lysozyme was dissolved at 5 mg/mL in 10 mM NaCl.

IgG, a human serum IgG-lyophilized fractionated purified (IRHUGGF-LY) was from Innovative Research, Inc., and had a purity reported as over 97%.

Proteinase K purified from the fungus Tritirachium album was obtained from Millipore Sigma. Proteinase K was dissolved at 20 mg/mL in pure 18.2 mΩ water.

Casein from bovine milk was obtained from Millipore Sigma, product no. 218680. Casein was dissolved at 10 mg/mL in pH 8 Tris under heating in an orbital shaker at 40 °C for 24 h.

### 2.2 Static and Dynamic Light Scattering

A Brookhaven Instruments NanoBrook Omni (Holtsville, New York) was used for DLS, using λ_ο_ = 640 nm as the vacuum wavelength of the vertically polarized incident light, and θ = 90° detection, with a fixed scattering vector magnitude of , where *n*_s_ = 1.333 is the index of refraction of water. Standard second-order cumulant analysis was used to yield the *z*-average diffusion coefficient and polydispersity index, *Q*, the ratio of the quadratic term of the logarithm of power series expansion of the electric field autocorrelation function to the square of the linear term. An improved method for polydispersity determination has been proposed.^22^ A second DLS instrument, a Brookhaven 90 Plus with λ_0_ = 640 nm, was also used.

SLS was carried out on a Fluence Analytics (now Yokogawa Fluence Analytics, Houston, Texas) Argen device, equipped with 16 independent sample cells, each with its own adjustable temperature and stirring, and incident laser source at λ_ο_ = 660 nm. Experiments are carried out simultaneously in as many as 16 independent sample cuvettes inserted into the sample cells.

### 2.3 Real-Time Dialysis Monitoring

**Scheme 1:**
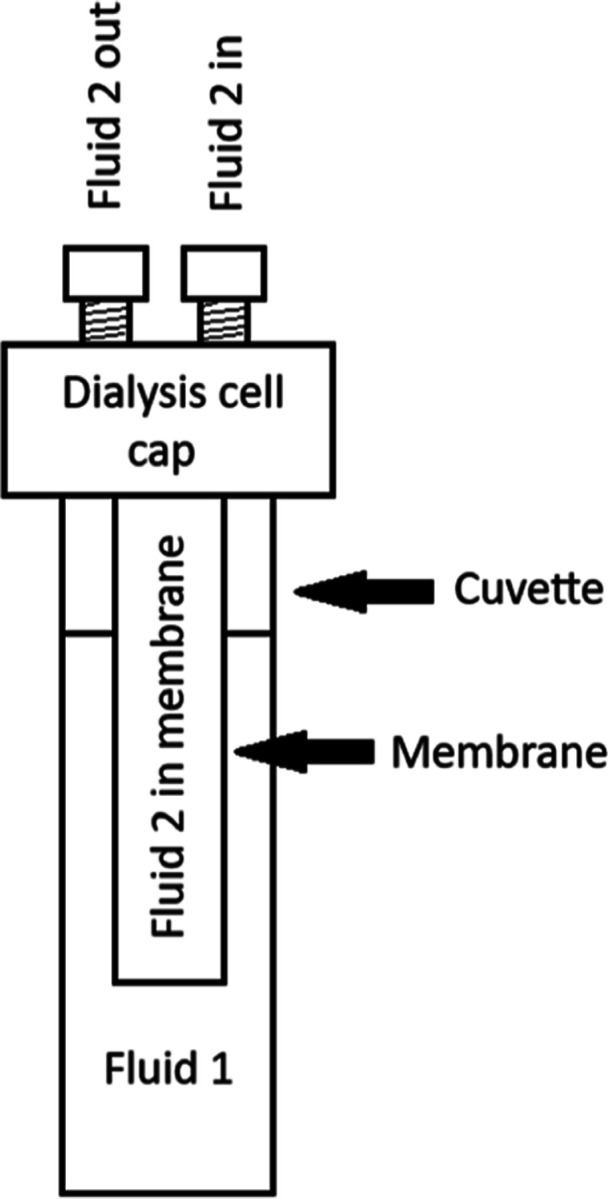
Basic Elements of the Spectroscopically Useful Dialysis Cuvette

A device was developed that provides a cap structure insertable into any 1 cm cuvette, hence allowing real-time monitoring of membrane-mediated processes, such as dialysis, in any optical instrument which accepts this type of standard cuvette, such as static and DLS, UV/visible absorption, fluorimeter, circular dichroism, etc. The dialysis cuvettes were used directly with Brookhaven DLS and Fluence Argen. The cap structure consists of a hollow cylindrical post around which a tubular membrane can be hermetically sealed, thus partitioning the fluid content of the cuvette into fluid 1, constant at 2.5 mL, which contains the macromolecule of interest, and fluid 2, which contains the dialysate. The cap is fitted with an inlet and outlet so that the dialysate can be circulated externally through a reservoir, which allows both proportioning the volumes of fluids 1 and 2, and accepting conductivity, pH, and other probes. Fluid 1 and any changes in the macromolecules due to dialysis are continuously monitored by whatever instrument the cuvette is placed into. Scheme 1 shows the general features of the dialysis cell.

In this work, NaCl and guanidine-HCl (Gd) were used as the simple electrolyte and chaotropic agent, respectively. Since NaCl and Gd both increase the conductivity of aqueous solutions, their concentration in fluid 1 could be computed by continuously measuring the conductivity of fluid 2. When dialyzing against 5 M NaCl or 6 M Gd fluid 2 was 100 mL, so that fluid 1 was at 4.88 M NaCl and 5.85 M Gd at the end of forward dialysis. In reverse dialysis to pure water, fluid 2 was at 1000 mL, so that at the end of reverse dialysis fluid 1 was at 0.00487 NaCl and 0.0058 M Gd. For gelatin and BSA, fluid 2 was at 500 mL for reverse dialysis against 10 mM aqueous NaCl and against phosphate (NaH_2_PO_4_–Na_2_HPO_4_) or Tris/Trizma buffer systems, respectively.

The dialysis membrane was Sigma-Aldrich cellulose D9277 with ∼10,000 g/mol molar mass cutoff.

The reader will notice that dialysis results are sometimes represented versus time and in other instances versus the concentration of the electrolyte, either [NaCl] or [Gd]. It is often more intuitively appealing to see the time course of the dialysis, whereas when inquiring into what concentrations of NaCl or Gd are associated with data trends, the representation versus [NaCl] or [Gd] can offer more insights.

It will be further noticed that different ranges of NaCl and Gd were used for different macromolecules, as shown in Table 1. It is important to note that dialysis scans were originally run up to 5 M NaCl and 6 M Gd for all the macromolecules and then the final value was narrowed in order to home in on the range of electrolyte concentrations that had the most effect on a given macromolecule. The data in the Results section are for the narrowed electrolyte range, not the initial full strength dialyses. For example, for PSS, the electrolyte induced changes in scattering are nearly essentially complete by 100 mM NaCl (Figure 2), whereas for BSA, the dramatic threshold effects occurred at 2 and 6 M Gd (Figure 11).

**Table 1:** Summary of Interactions and Behavior for the Macromolecules Monitored during Dialysis^a^

	interactions			NaCl			Gd		
polymer	E	A	HP	[NaCl]_max_	behavior	rev	[Gd]_max_	behavior	rev
PSS	Y	N	N	0.1 M	PE	yes	0.1 M	PE	yes
alginate	Y	N	Y	2.5 M	aggregative/HP	no	3.8 M	PE/dissociative/HP	no
gelatin	Y	Y	Y	5 M	PE/PA/HP	no	6 M	PE/dissociative/HP	no
BSA	Y	Y	Y	4 M	PE/HP	NA	6 M	sharp aggregation threshold	no
lysozyme	Y	N	Y	1 M	dialysis hindered by time-dependent aggregation	no	1 M	no change	no change
IgG				NA	NA	NA	6 M	PE/HP/associative/dissociative	partial
proteinase	Y	N	Y	4 M	PE/HP/associative	no	6 M	HP/dissociative	no
casein	Y	Y	Y	4 M	Complex	no	6 M	complex	no

a E = Polyelectrolyte potential. A = Polyampholyte potential. HP = hydrophobic and/or H-bond effects. PE = Polyelectrolyte. PA = Polyampholyte. NA = data not available.

### 2.4 Monitoring at Fixed [NaCl] and [Gd] for Time-Dependent Processes

As in all ramped methods (e.g., differential scanning calorimetry), it is important that any time-dependent processes occurring in the samples be faster than the ramp rate, so that the system is instantaneously in equilibrium at each point during the temporal ramp. Otherwise, the time-dependent process in the sample can be convolved with the time-dependent ramping procedure, which can cloud data interpretation. Accordingly, when evidence of time-dependent effects on the time scale of the dialysis was found, complementary time-dependent measurements were also made at fixed [NaCl] and [Gd].

### 2.5 Interpretive Model for Polyelectrolyte, Polyampholyte, and H-Bond/Hydrophobic Effects

The trends in the results of dialysis for the various types of macromolecules can be interpreted with a basic dimensionless model combining intermacromolecular potential energies of repulsive polyelectrolyte interactions (E), of attractive polyampholyte interactions (A), and of attractive H-bond/hydrophobic (HP) effect potential energies, which also subsume entropy changes, especially of the solvent. The object here is to make plausible forms of net potential energies under different interplays of E, A, and HP for chaotropic agents (Gd here) and E and A for simple electrolytes (NaCl here). When the net potential energy is positive, no association or aggregation is expected to occur, and if aggregates or associations are present, the positive potential may lead to their dissociation. When the net potential is negative, associations or aggregates can be formed. For purposes of terminology, “aggregate” will refer here to irreversible associations of macromolecules, whereas “associations” among macromolecules will be considered reversible. Since the focus in this work is on intermacromolecular processes, the dimensionless model does not include intramolecular potential energies. These can be added in future development as the need arises.

In the results below, the use of dialysis of the macromolecules against NaCl and Gd can help separate out which effects are operative and dominant over different concentration regimes. The idea of the dimensionless model is to make the interpretation plausible and not to make fits to the data. Further work can put absolute values on the potential energies. As an indication as to magnitudes, the electrostatic potential energy of two unscreened elementary charges separated by a distance of 1 nm is approximately 2.3 × 10^–19^*J* ≈ 1.4 eV, where eV = electronvolt.

The model starts by allowing macromolecules to interact by all three effects: E, A, and HP. The polyelectrolyte (E) term considers that the macromolecules each have a net charge and hence repulsive interactions expressed by a positive screened electrostatic potential energy, *U*_el,E_. The polyampholyte potential energy, *U*_el,A_, is the negative screened potential energy between opposite charges of the interacting chains. The model then posits a negative interchain potential energy due to possible H-bonds and HP effects, *U*_HP_, but does not attempt to distinguish between the two types of attractive interactions at this level of development since Gd can suppress both H-bond and HP effects.

When dealing with a nonchaotropic salt, such as NaCl, only *U*_el,E_ and *U*_el,A_ vary with ionic strength, whereas *U*_HP_, which is negative, will remain constant. When a chaotropic salt, such as Gd, is considered, the *U*_HP_ decreases as [Gd] increases.

For a charge *q* of a spherical object of radius *R*, the screened electrostatic potential of a charge ϕ_el_(κ,*r*) at distance *r* is given (in volts) by
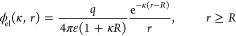
1where *r* is the distance from the center of the charge. ϕ_el_(κ,*r*) is positive for ϕ_el,E_ and negative for ϕ_el,A_, and κ is the Debye screening parameter, given by (in MKS units)
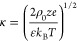
2where ρ_0_ is the charge density (C/m^3^) due to the bulk concentration of added electrolyte, which is either Gd or NaCl in the current case (ρ_0_ is the charge density of positive charge, which of course is equal and opposite in sign to the negative charge density that reflects electroneutrality in the simple bulk electrolyte solution), *e* is the elementary charge, *z* is the valence for symmetric electrolytes (*z* = 1 for both Gd and NaCl), ε = ε_0_*D*, where ε_0_ = 8.85 × 10^–12^ C^2^/N m^2^ is the permittivity of free space and *D* is the solution dielectric constant (∼80 for H_2_O at STP), *k*_B_ is Boltzmann’s constant (1.38 × 10^–23^ J/K), and *T* is the temperature in Kelvin (κ is only weakly dependent on *T* near room temperature). Here, ρ_0_ is proportional to the ionic strength of the electrolyte [IS] (mol/L)
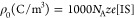
3

[IS] is substituted by [Gd] and [NaCl], in their respective cases.

The screened electrostatic potential energy between two interacting charges has a more complex expression^23,24^ and so will be synthesized here into an average dimensionless potential energy ⟨*U*_el_⟩. This potential energy is the electrostatic potential energy, Boltzmann-averaged over all interaction distances between charges. The net electrostatic potential energy ⟨U_el,net_⟩ is composed of the sum of the similarly spatially Boltzmann-averaged positive polyelectrolyte potential energy ⟨U_el,E_⟩ and negative polyampholyte potential energy ⟨U_el_,_A_⟩
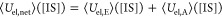
4where [IS] is the ionic strength from any simple electrolyte, regardless of symmetry and valence, and includes the symmetric, monovalent Gd and NaCl. Because the distance has been averaged out in the dimensionless potential energies, the E and A potential energies can be simplified to
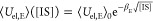
5a
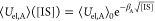
5bwhere  is the positive potential energy between two unscreened charges of the same sign (i.e., between two polyelectrolyte chains), and  is the negative potential energy between two unscreened charges of opposite sign. β_Ε_ and β_A_ subsume the factors connecting κ and [IS] in eqs 2 and 3, and the Boltzmann-averaging over distance. β_Ε_ and β_A_ are different because the intercharge interactions occur on different length scales, i.e., distances between net charged polyelectrolyte chains in β_Ε_ and distance between opposite sign charges interacting closely between chains. According to eqs 5a and 5b the polyelectrolyte and polyampholyte potential energies have different signs, magnitudes, and spatial variations.

In this treatment, the intrachain charge–charge interactions and HP interactions are ignored because the main features for the biomacromolecules under dialysis in this work are governed by interchain association, dissociation, and aggregation.

Now, ⟨*U*_HP_⟩ ([IS]) represents averaged H-bond and HP associations and may be represented by a sigmoidal type cooperative binding expression for Gd
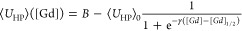
6awhere  is a negative potential energy, and which is independent of [NaCl]. For NaCl ⟨*U*_HP_⟩ is constant
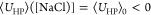
6bwhere the negative  is the HP potential energy at [Gd] = 0, [Gd]_1/2_ is the concentration of Gd at which the sigmoidal energy is at its half-value, γ controls the rate at which increasing [Gd] diminishes the H-bond/HP interaction (temperature = 25 °C was not varied in the dialysis procedure and so the *T*-dependence of *U*_hp_ is not explicitly shown). *B* is a constant given by
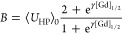
7which ensures that
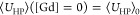
8

The average net energy of the system, ⟨*U*_net_⟩ ([Gd]) is just the sum of the electrostatic and HP potentials. The net potential energies are then
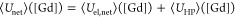
9a
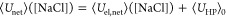
9b

The behavior of ⟨*U*_net_⟩([Gd]) and ⟨*U*_net_⟩([NaCl]) in this model should determine whether interacting macromolecules dissociate or remain dissociated, associate, or aggregate. In developing biologic drug formulations, the concentration regimes of electrolytes and other excipients can be determined so as to optimize the stability of the formulations.

## 3 Results

### 3.1 Synthetic Polyelectrolyte, PSS; E Interactions Only at Low Ionic Strength

PSS resembles a negatively charged random coil in solution, whose coil dimensions and repulsive interactions with other coils are sensitive to ionic strength. The effects of ionic strength on coil size and interchain interactions should manifest during dialysis, and one effect may dominate over the other. There is no secondary structure in PSS, so dialysis with Gd should not be substantially different from that with NaCl.

**Figure 1:**
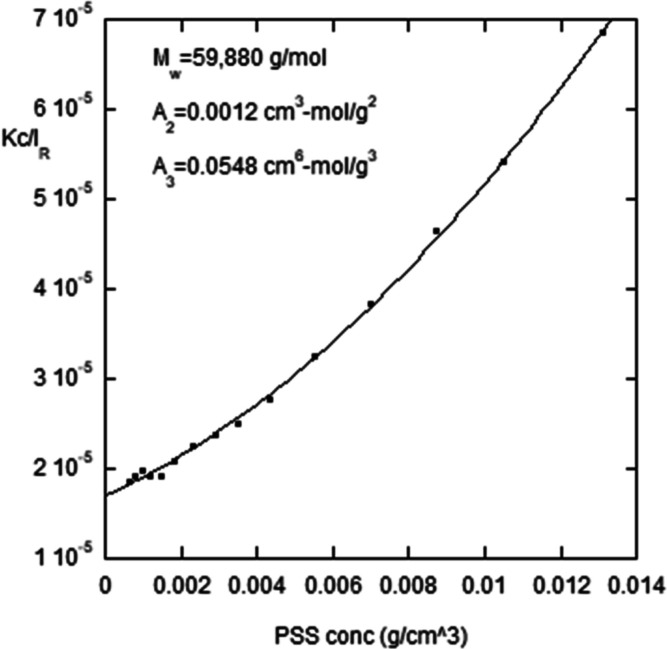
*Kc*/*I*_R_ vs [PSS] for PSS in 100 mM NaCl, with corresponding values of *M*_w_, *A*_2_, and *A*_3_, obtained by the quadratic fit to the data.

Figure 1 shows a Debye plot of PSS in 100 mM NaCl. PSS is a small macromolecule with apparent hydrodynamic diameter (see eqs 16 and 17 for definition) *d*_H,ap_ ≤ 12 nm, which is much smaller than the 640 nm (DLS) and 660 nm (SLS) of incident light, so that DLS and SLS measurements should have no significant angular dependence, and measurements at θ = 90° yield *q*^2^ ⟨*S*^2^⟩_*z*_/3 ≪1, and the *q* = 0 expression can be used
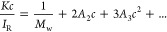
10where *c* is the polymer mass concentration (g/cm^3^), *I*_R_ is the Rayleigh scattering ratio (1/cm), and *K* is an optical constant, given for vertically polarized incident light by
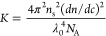
11where *n*_s_ is the index of refraction of the aqueous medium (1.333), λ_0_ = 640 (DLS) or 660 nm (SLS) is the vacuum wavelength of the incident light, *N*_A_ is Avogadro’s number, and d*n*/d*c* = 0.185 cm^3^/g is the incremental index of refraction of PSS in the aqueous medium.

Fitting the data in Figure 1 with a quadratic function in concentration, eq 10, yields weight-average molar mass, *M*_w_ = 58,980 g/mol, and at 100 mM NaCl, the second virial coefficient *A*_2_ = 0.0012 cm^3^ mol/g^2^, and third virial coefficient *A*_3_ = 0.0548 cm^6^ mol/g^3^.

For random coils, *A*_3_ is related to molar mass *M* by
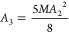
12

*A*_3_ from the quadratic fit term in Figure 1 gives *A*_3_ = 0.0548 cm^6^ mol/g^3^. *A*_3_ computed by the theoretical eq 12, using the values of *M*_w_ and *A*_2_ in the figure, *A*_3_ = 0.0539 cm^6^ mol/g^3^, is in remarkable agreement with the prediction. Furthermore, when the scattered intensity is plotted versus *c*, there is a maximum intensity that occurs at *c*_m_, after which the intensity decreases due to *A*_3_ as *c* increases above *c*_m_. The relationship between *c*_m_ and *M* is
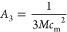
13

For the data of Figure 1, *c*_m_ = 0.0105 g/cm^3^, which yields *A*_3_ = 0.0505 cm^6^ mol/g^3^, by eq 13, using *M* = *M*_w_, which is in good agreement with both the quadratic fit to the data of Figure 1 and the theoretical expression of eq 12.

**Figure 2:**
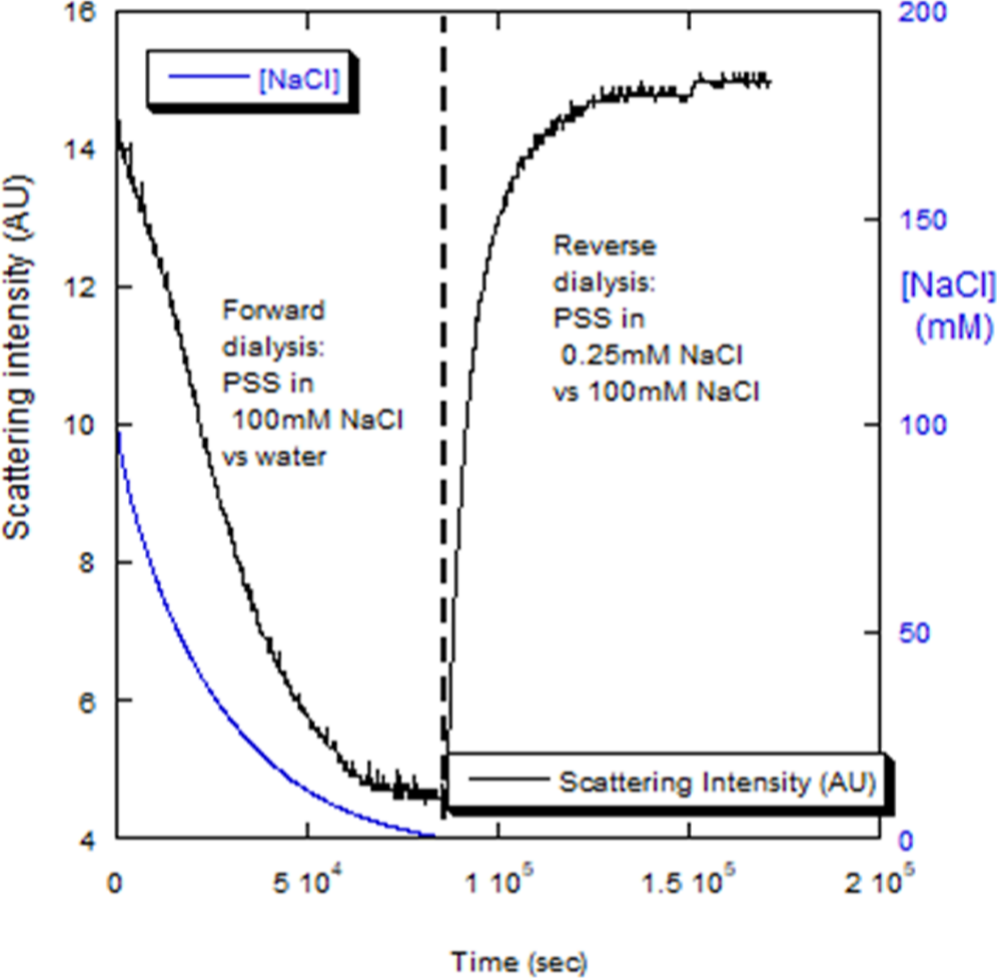
Complete dialysis cycle for 0.00326 g/cm^3^ PSS starting in 100 mM [NaCl] against pure water, and the reverse portion from 0.25 mM NaCl to 100 mM NaCl. [NaCl] in the PSS solution is also shown for the forward dialysis.

Figure 2 up to 87,000 s shows the scattering intensity (arbitrary units) versus time as PSS at 0.00327 g/cm^3^ in 100 mM NaCl aqueous solution is forward dialyzed against pure water. Fluid 1 contained 2.5 mL of PSS solution, and the pure water dialysate comprised a reservoir of 1000 mL. Also shown is [NaCl] in fluid 1 during this forward cycle. After 87,000 s, the PSS solution in fluid 1, now at 0.25 mM NaCl, is dialyzed against 100 mM NaCl in fluid 2.

Two immediate observations can be made regarding the complete dialysis cycle.

1) The dialysis process is fully reversible, i.e., PSS returns to its original scattering value after the complete cycle (the return intensity is slightly higher than the initial due to osmotic pressure draining a small amount of the 2.5 mL of fluid 1 over the course of the dialysis, thus slightly concentrating the PSS)
2) As expected for a polyelectrolyte, the scattering intensity decreases as the ionic strength ([NaCl]) decreases. This is due to the deshielding of the anionic sulfate groups, which causes the polyelectrolyte coil to expand, while simultaneously increasing the electrostatically enhanced mutual excluded volume *V*_ex_, and hence decreasing scattered intensity by eq 1, because *A*_2_ increases as

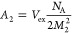
14

The importance of the *A*_3_ term on scattered intensity increases as *A*_2_ increases with decreasing [NaCl]. This is seen by the ratio of the second to third terms in eq 10; the smaller the value, the greater the effect of *A*_3_ compared to *A*_2_. By using eq 12, which holds well in Figure 1, the ratio can be expressed as
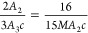
15

**Figure 3:**
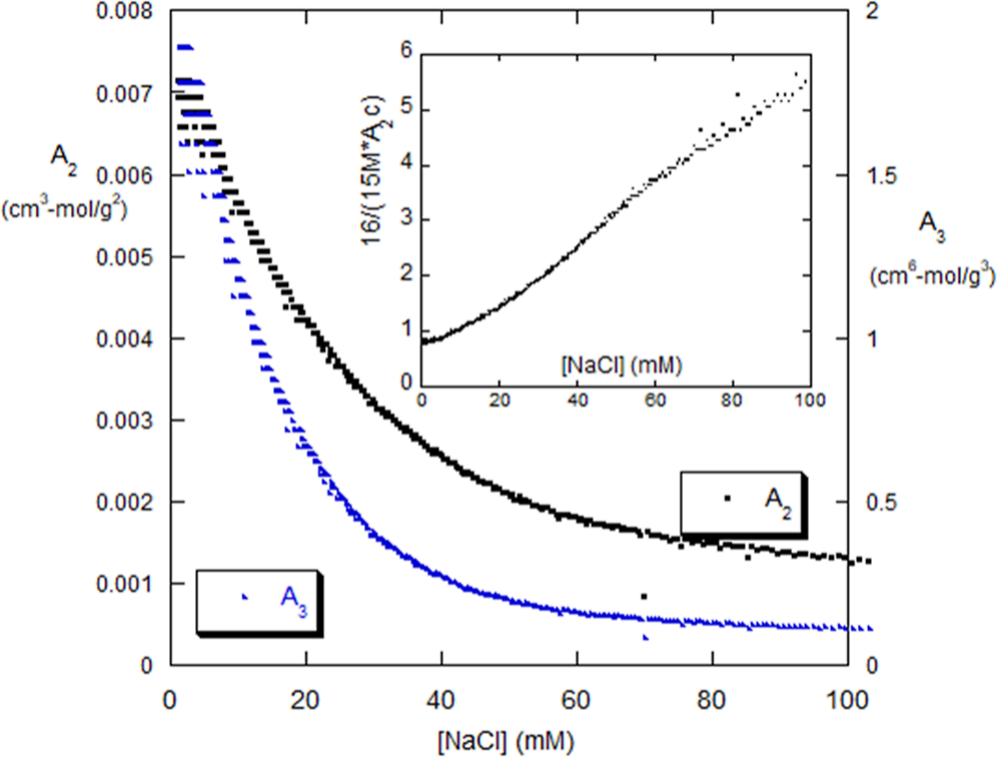
*A*_2_ and *A*_3_ versus [NaCl]. The inset shows the ratio of 2*A*_2_/*A*_3_*c*, which measures the relative importance of the *A*_2_ effect to the *A*_3_ effect.

The ratio runs from 5.4 at 100 mM NaCl to 0.77 at 0.25 mM NaCl. Equation 12 can be used to convert eq 10 into a quadratic equation in *A*_2_. Solving for *A*_2_ using the *Kc*/*I*_R_ data for the forward dialysis, and then computing *A*_3_ by eq 12 yields the behavior for *A*_2_ and *A*_3_ shown in Figure 3. The inset to Figure 3 shows the ratio of the *A*_2_ to *A*_3_ term in eq 10, as given by eq 15. At high [NaCl], the *A*_2_ effect is much larger than the *A*_3_ effect, but the effects become roughly equal at low [NaCl].

**Figure 4:**
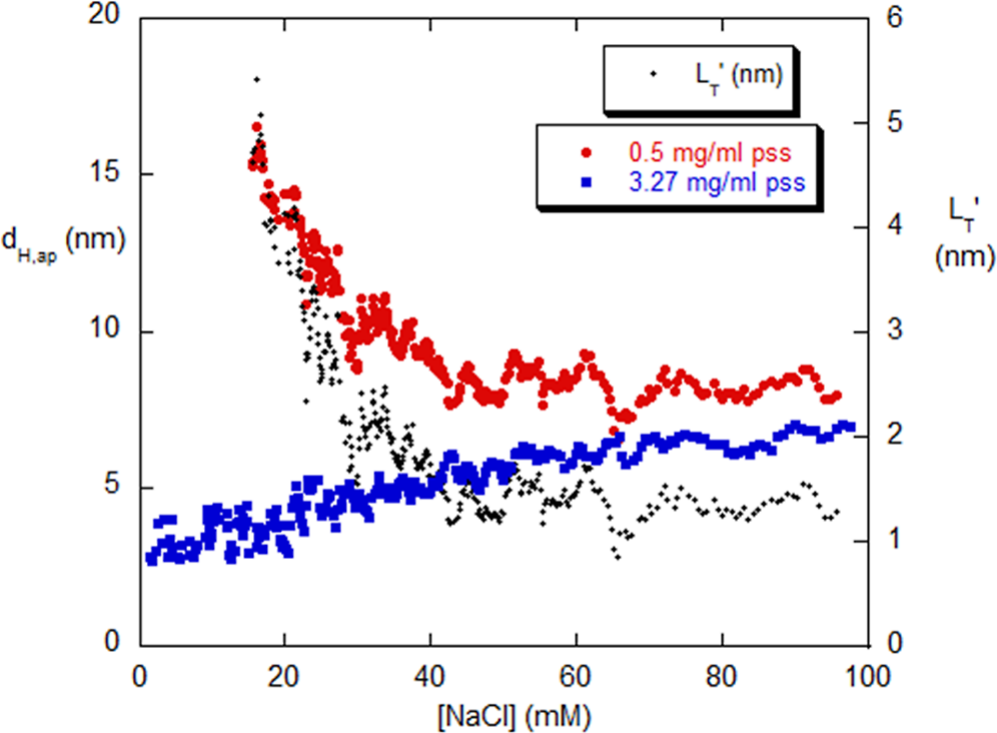
*d*_H,ap_ obtained from ⟨*D*⟩_*z*_ by eqs 16 and 17. At 3.27 mg/mL PSS, the interchain hydrodynamic parameter *k*_D_ dominates *d*_H,ap_, whereas at 0.50 g/mL, the coil shrinkage with increasing [NaCl] dominates. *d*_H,ap_ for both concentrations converges as 100 mM [NaCl] is approached. Shown on the right-hand scale is the apparent persistence length, *L*_T_′.

The DLS results are shown in Figure 4 for the apparent hydrodynamic diameter *d*_H,ap_. DLS measures the *z*-averaged translational diffusion coefficient, ⟨*D*⟩_*z*_, which is related by the Stokes–Einstein equation to the *z*-average reciprocal hydrodynamic diameter 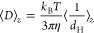
16where η is the solution viscosity. The “hydrodynamic diameter” reported by most DLS instruments is an “apparent hydrodynamic diameter”, *d*_H,ap_, which is the reciprocal of the *z*-averaged reciprocal hydrodynamic diameter , that is
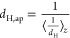
17

The trend in Figure 4 for PSS at 3.27 mg/mL is that *d*_H,ap_ increases with [NaCl]. There are two effects that can occur concerning ⟨*D*⟩_*z*_, and correspondingly for *d*_H,ap_: (i) the polyelectrolyte coil should shrink as [NaCl] increases, so that *d*_H,ap_ decreases and (ii) the hydrodynamic interaction parameter *k*_D_ should decrease as increasing [NaCl] weakens the interpolymer interactions so that *d*_H,ap_ increases. The interaction effect, expressed to the first order is
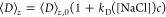
18where *k*_D_ ([NaCl]) explicitly shows that *k*_D_ depends on [NaCl], *c* is the polymer concentration (g/cm^3^), and ⟨*D*⟩_*z*,0_ is the *z*-averaged diffusion coefficient extrapolated to *c* = 0 at high [NaCl].

Figure 4 shows that the latter effect (ii) dominates at 3.27 mg/mL PSS. At very low [NaCl], the scattered intensity was low and erratic, leading to large fluctuations in ⟨*D*⟩_*z*_ and polydispersity (from the second cumulant of the autocorrelation function expansion).

It is interesting to note that at very low [NaCl], there is no “slow mode of diffusion” (i.e., low ⟨*D*⟩_*z*_ at very low [NaCl]), as has often been reported^25,26^ but which has also been given an alternative interpretation;^27^ at very low ionic strength, the well-dissolved polyelectrolyte scatters very little light, allowing even small amounts of aggregates or impurities to erratically dominate the weak scattering and yield anomalously low diffusion coefficients (“the stars come out at night”).^28^

Figure 4 also shows that effect (i) dominates at 0.50 mg/mL PSS; the polyelectrolyte coil shrinks as [NaCl] increases. This is in contrast with the opposite trend at 3.27 mg/mL where effect (ii) dominates; the hydrodynamic interaction parameter *k*_D_ should decrease as increasing [NaCl] weakens the interpolymer interactions so that *d*_H,ap_ increases. Figure 4 also shows that *d*_H,ap_ converges to about 8.2 nm for both PSS concentrations as [NaCl] approaches 100 mM as the relatively high IS leads to maximum coil shrinkage while reducing *k*_D_ to a negligible effect. The low [NaCl] *d*_H,ap_ of 15 nm for this PSS of 59 880 g/mol scales via *d*_H,ap_ ∼ *M*^0.6^ to 6.2 nm, the value found for the case of PSS of 15,800 g/mol in a reference detailing many solution properties of PSS.^29^

Treating PSS as a semiflexible polyelectrolyte allows the total persistence length *L*_T_ to be expressed as *L*_T_ = *L*_0_ + *L*_e_, where *L*_0_ is the intrinsic persistence length and *L*_e_ is the ionic strength-dependent electrostatic persistence length, in the absence of long-range excluded volume interactions.^30^ The wormlike chain model connects *L*_T_ to the mean-square radius of gyration in the absence of excluded volume, , via the contour length L of the polyelectrolyte chain.^31^ In the random coil limit (*L* ≫ *L*_T_), the model gives
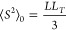
19a

The inclusion of positive long-range excluded volume on increasing  beyond the expression of eq 19a is a difficult theoretical subject,^32^ and light scattering measurements give the net ⟨*S*^2^⟩, not the value in the absence of excluded volume. A practical means of avoiding the complexities is to use eq 19a, substituting the measured ⟨*S*^2^⟩^1/2^ and an apparent total persistence length, *L*_T_′^33^
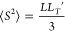
19b

PSS has 207 g/mL per monomer contour length of 0.256 nm, so that *L* = 74 nm for *M*_w_ = 59,800. For nondraining random coils,^34^*d*_H,ap_ ∼ 1.4 ⟨*S*^2^⟩^1/2^.

The right-hand *y*-scale of Figure 4 shows *L*_T_′ for PSS, based on the 0.5 mg/mL data for *d*_H,ap_ in Figure 4, computed according to
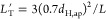
20

*L*_T_′ varies from 1 nm at high [NaCl] to 5 nm at low [NaCl] (justifying the use of the coil limit). This range of persistence lengths is typical for polyelectrolytes without any secondary or tertiary structure.

**Figure 5:**
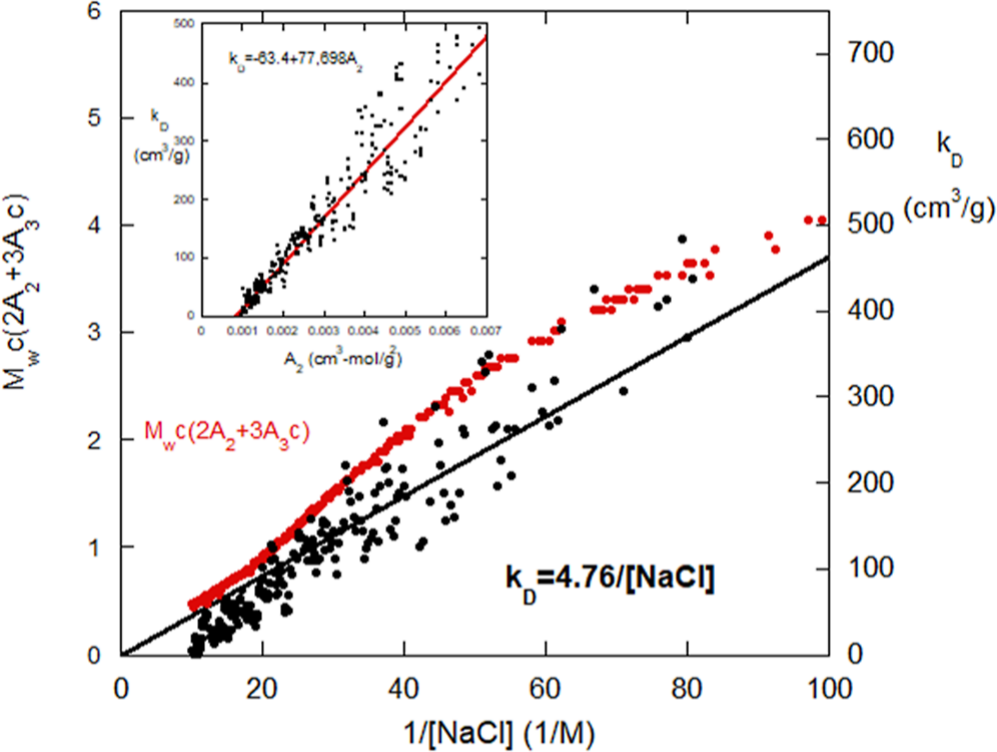
Hydrodynamic interaction parameter *k*_D_ versus [NaCl] (mM) for PSS. Also shown is the dimensionless ratio of the combined *A*_2_ and *A*_3_ effects divided by the 1/*M*_w_ in eq 10. The inset shows a linear relationship between *k*_D_ and *A*_2_.

Figure 5 shows *k*_D_([NaCl]) versus 1/[NaCl] obtained from eq 18 by
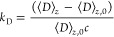
21*k*_D_ becomes quite large as [NaCl] decreases. This is to be expected since the interchain repulsion increases as [NaCl] decreases, and the scattering becomes increasingly dominated by the *A*_2_ and *A*_3_ interchain interaction terms. The linear behavior of *k*_D_ versus 1/[NaCl] is in agreement with the theoretical result of Imai and Mandel,^35^ and the experimental results of Tanahatoe and Kuil.^36^Figure 5 also shows the dimensionless ratio of the *A*_2_ and *A*_3_ terms to the *M*_w_ term, given by
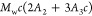
22

Figure 5 shows that, whereas the *M*_w_ term dominates at high [NaCl], the combined *A*_2_ and *A*_3_ effects rapidly grow significantly larger than the *M*_w_ term as [NaCl] decreases. The inset of Figure 5 shows *k*_D_ versus *A*_2_. It is frequently claimed that *k*_D_ is a sort of ‘hydrodynamic *A*_2_′ and should be functionally related. The inset of Figure 5 shows an essentially linear relationship between *k*_D_ and *A*_2_.

Dialysis of PSS in pure water against Gd showed the same type of ionic strength behavior as that with NaCl over the same concentration range and was fully reversible.

### 3.2 Natural Polyampholyte: Gelatin; Combined E, A, and HP Interactions

**Figure 6:**
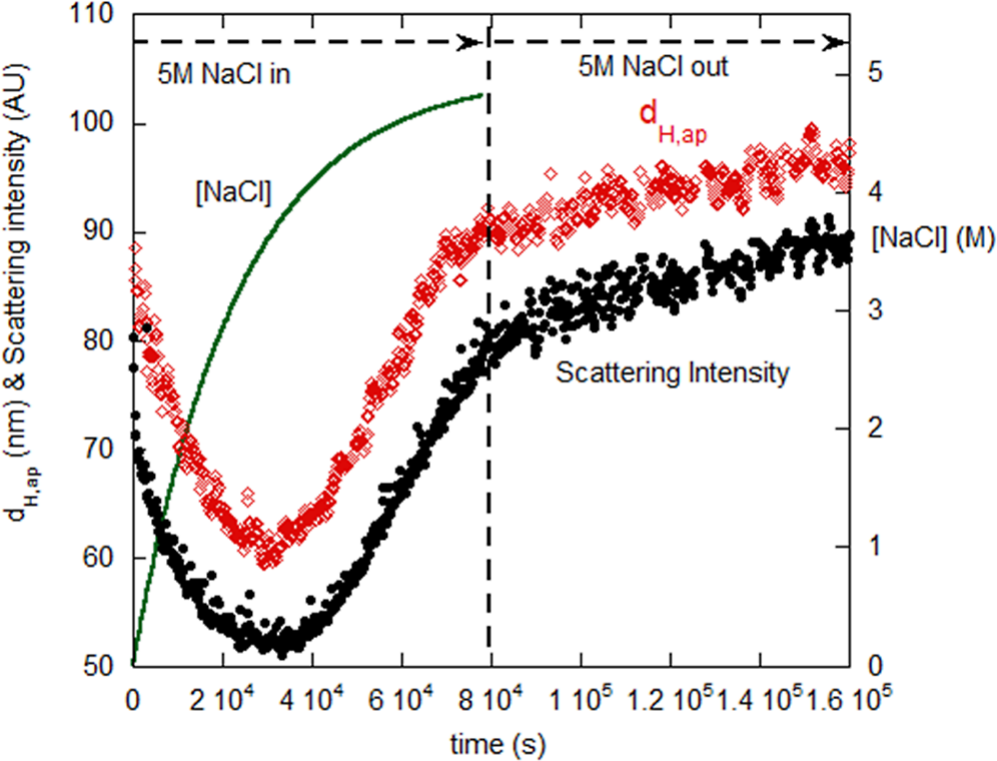
Apparent hydrodynamic diameter *d*_H,ap_ and scattering intensity (AU) for forward dialysis of 0.001 g/cm^3^ gelatin in 10 mM NaCl against 5 M NaCl, for zero up to 80,000 s. Reverse dialysis against water is shown from 80,000 to 160,000 s. Also shown is [NaCl] for the forward dialysis.

**Figure 7:**
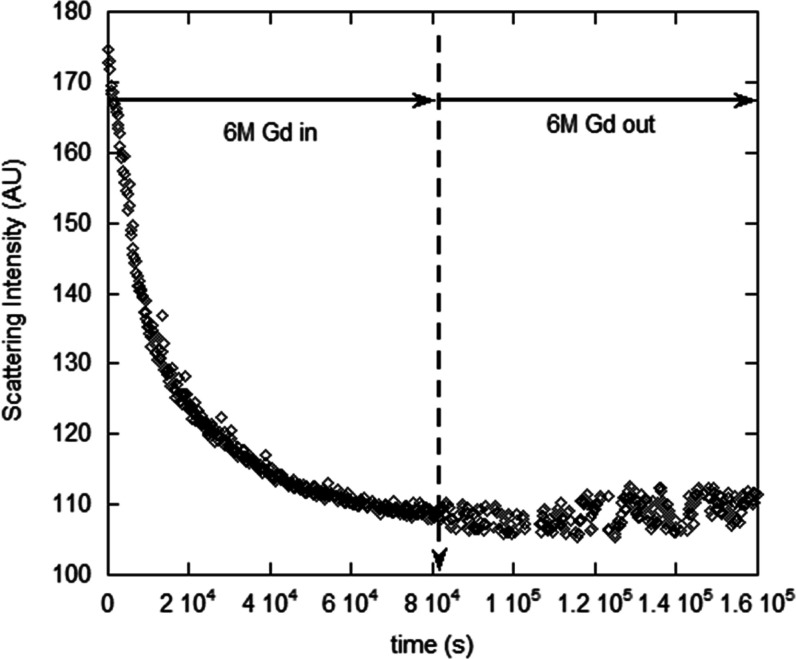
*d*_H,ap_ for dialysis of gelatin in 10 mM NaCl from water against 6 M Gd, and reverse.

Figure 6 shows scattering intensity (AU) and *d*_H,ap_ for gelatin initially in aqueous 10 mM NaCl and dialyzed against 5 M NaCl, and for reverse dialysis against pure water. [NaCl] is also shown for forward dialysis. *d*_H,ap_ closely follows the form of scattering intensity. Figure 7 shows scattering intensity for gelatin dialysis against 6 M Gd and reverse against 10 mM NaCl (*d*_H,ap_ was also steady, not shown). Fluid 1 was 2.5 mL, and Fluid 2 was 100 mL for the forward dialysis and 500 mL for the reverse dialysis. Hence, at the end of the forward dialysis [NaCl]_forward,final_ = 4.87 M, and [Gd]_forward,final_ = 5.85 M, and for the reverse dialysis [NaCl]_reverse,final_ = 0.020 M, and [Gd]_reverse,final_ = 0.029 M.

The data are described as follows: in pure water, because there are negative and positive charges on the polymer chains, there is some initial association of chains in pure water due to polyampholyte attraction between positive and negative charges. As ionic strength increases initially for both NaCl and Gd, the opposite charges are shielded from each other, the attractive polyampholyte potential energy ⟨*U*_el,A_⟩ decreases, ⟨*U*_net_⟩ becomes positive, and the chains dissociate. In NaCl, after about 2.8 M [NaCl], the chains begin to reassociate, as seen in the increasing intensity and *d*_H,ap_ in Figure 6. These arrive at a final value, and these reassociated chains are not reversible, when dialyzing back against pure water, and so are considered irreversible aggregates. In Gd, there is likewise a dissociation as [Gd] increases, but there is no reassociation all the way up to 6 M Gd, and the chains remain dissociated upon reversal. In the Gd case, the disassociation is irreversible.

These effects can be conceptually understood by the interpretive model above. Since Gd can interrupt both H-bonds and suppress the HP effect, it follows that one or both of these effects is involved in the reassociation of chains in NaCl. This follows because the reassociation does not occur in Gd, which suppresses these HP interactions. In NaCl, the conjecture is that as [NaCl] initially increases, the attractive polyampholyte electrostatic associations are suppressed and allow dissociation of chains as ⟨*U*_net_⟩ becomes positive. As further shielding by NaCl occurs, they get close enough to each other that the HP and/or H-bond forming portions of the chains can form associations and ⟨*U*_net_⟩ becomes negative. These associations are stronger than the electrostatic associations and so the chains remain irreversibly associated with each other even as [NaCl] decreases during reverse dialysis. This suggests that the initial associations among chains are nearly wholly electrostatic and that the association at high [NaCl] is due to HP and/or H-bond effects.

The interpretive model considers each of the initial gelatin polymer chains as being both a polyelectrolyte with a net charge and a polyampholyte, which interact with each other via electrostatic potential energies, ⟨*U*_el,E_⟩ and ⟨*U*_el,A_⟩, and H-bond/HP potential energies, ⟨*U*_HP_⟩. For a “generic” gelatin, p*K*_a_ ∼ 4.7, so that in the unbuffered solution, the polymers should have a net negative charge, giving it repulsive polyelectrolyte properties in addition to attractive polyampholyte interactions due to mixed positive and negative charge groups in the gelatin amino acids. Furthermore, gelatin can associate via HP interactions.

**Figure 8:**
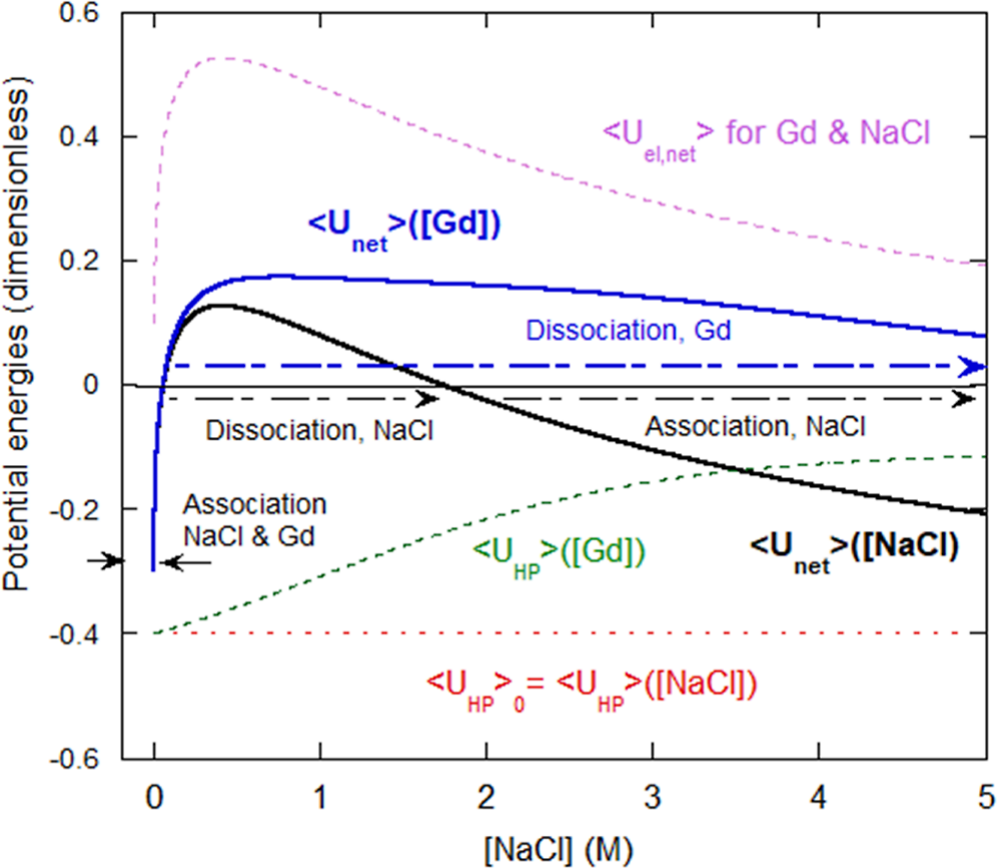
Net energies *U*_net_ for gelatin versus [NaCl] and [Gd]. Both start negative but quickly become positive, leading to dissociation of chains. *U*_net_ remains positive for Gd, and so the chains continue to dissociate and never reassociate. In contrast, *U*_net_ for NaCl drops back to negative as [NaCl] increases, leading to a reassociation of gelatin chains. The constituent potentials are shown with dashed lines.

Figure 8 shows dimensionless ⟨*U*_net_⟩([Gd]) and ⟨*U*_net_⟩([NaCl]), from eqs 9a and 9b, using the following parameters; β_Ε_ = 1 nm, β_A_ = 2 nm,  = 2,  = −1.9, ⟨*U*_hp,0_⟩ ≥ −0.40, γ = 1 (1/Μ), [Gd]_1/2_ = 1 M, κ(1/nm) ∈ {0, 2.5}, and [IS] = κ^2^. ⟨*U*_el,net_⟩ is also shown and is the same for NaCl and Gd. ⟨*U*_HP_⟩ is also shown for both NaCl, for which ⟨*U*_HP_⟩ is constant, and for Gd, for which ⟨*U*_HP_⟩ diminishes sigmoidally.

Figure 8 shows that ⟨*U*_net_⟩ < 0 at very low [IS], so there is interchain association. At low [IS] during forward dialysis, with both NaCl and Gd, ⟨*U*_net_⟩ quickly becomes positive, with the onset of chain dissociation. As [NaCl] increases, ⟨*U*_net_⟩([NaCl]) goes negative, due to the unchanging, negative ⟨*U*_HP_⟩([NaCl]), and so chains begin to reassociate and continue to reassociate until the end of forward dialysis at 5 M NaCl. Upon reverse dialysis against water, the associations are irreversible and show a slow further increase in time.

In contrast to NaCl, as [Gd] increases during forward dialysis, ⟨*U*_HP_⟩([Gd]) weakens, and ⟨*U*_net_⟩([Gd]) remains positive throughout, so that dissociation continues. Upon reverse dialysis, the chains remain unassociated. This behavior is captured over a wide range of the above parameters, and no attempt is made here to set limits on or find fit values for each parameter.

### 3.3 Natural Polyelectrolyte: Alginate. E and HP Interactions

**Figure 9:**
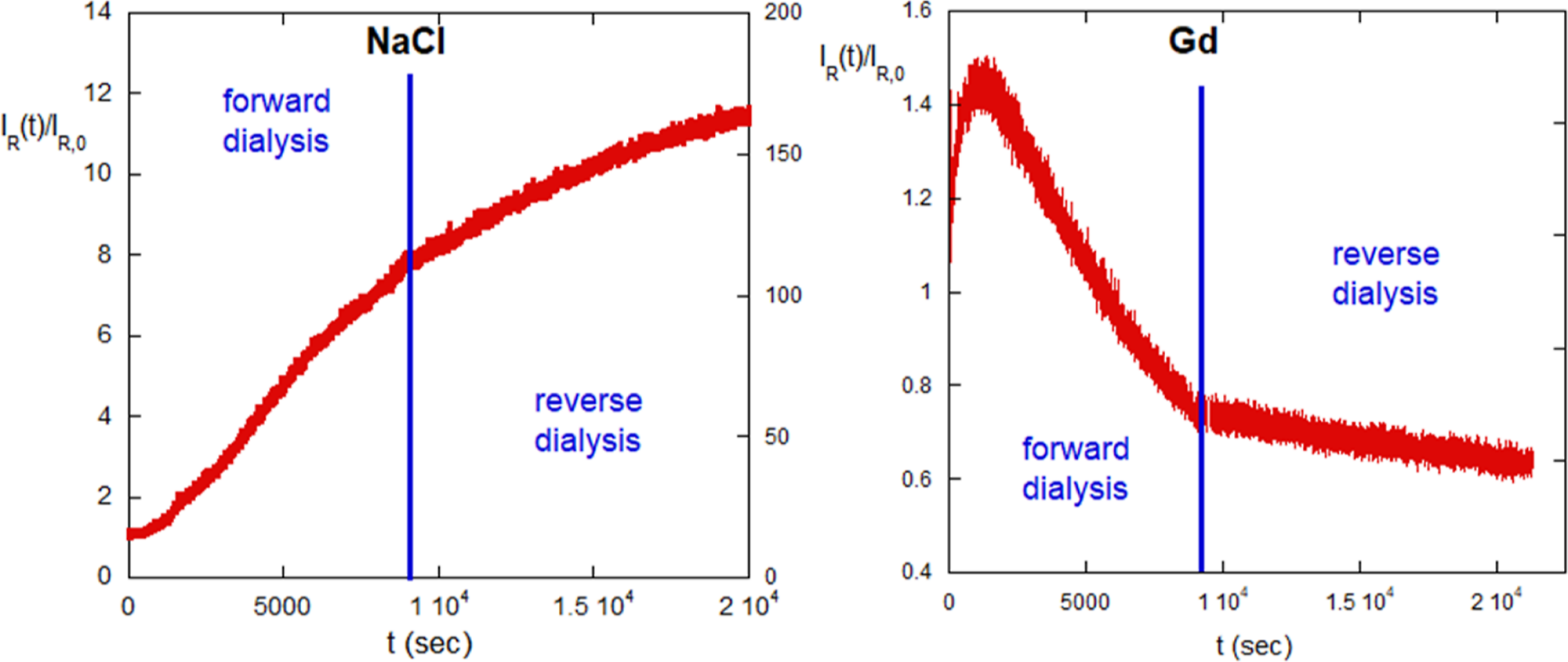
(a) Alginate under forward dialysis from water against 4 M NaCl, stopping at 2.5 M NaCl, and reverse. (b) Alginate under forward dialysis from water against 6 M Gd, stopping at 3.8 M Gd, and reverse.

Alginate in its sodium form is a polyanionic polysaccharide. Figure 9 shows the scattering intensity behavior for 0.001 g/cm^3^ alginate dialyzed from pure water against 4 M NaCl, and the reverse dialysis against pure water after the alginate was at 2.5 M NaCl. The data are represented as *I*_R_(*t*)/*I*_R,0_, where *I*_R_(*t*) is the scattering at time *t*, and *I*_R,0_ is the initial scattering at *t* = 0. The initial increase is the expected polyelectrolyte effect, as for PSS above, but after passing through an inflection point early on, at about 1300 s, it becomes clear that the subsequent scattering increase is due to an association process, which continues throughout the forward dialysis and then continues unabated during reverse dialysis against water. This suggests that the association process is driven by HP effects and forms aggregates of increasing size, whose ability to further associate under HP effects remains, even as [NaCl] is dialyzed away. The associations are, hence, irreversible aggregates.

Figure 9 shows the scattering intensity behavior for 0.001 g/cm^3^ alginate dialyzed from pure water against 6 M Gd, and the reverse dialysis against pure water after the alginate was at 3.8 M Gd. The difference from that in Figure 9 is striking. There is an initial rise in scattering, similar to Figure 9, due to the polyelectrolyte effect, but then a maximum in scattering is reached and then decreases monotonically for the rest of the forward dialysis to a value lower than its starting value in water. During the reverse dialysis, there is a continued decrease in scattering but at a highly reduced rate. By the end of the reverse dialysis, the scattering is less than that at the beginning of the dialysis cycle. This suggests that there were already associations between alginate chains originally, in pure water, and that the Gd both dissociates these as well as aggregates formed in the very early phase of forward dialysis, where intensity increases, before peaking and decreasing as [NaCl] increases further.

The behavior of Figure 9a,b can be interpreted by the above combined electrostatic and HP interactions. Now, however, there is no polyampholyte term, so ⟨*U*_el,A_⟩ ≥ 0, while ⟨*U*_el,E_⟩ is the polyelectrolyte term due to the negatively charged alginate chains.

There is a substantial biochemical difference between gelatin and alginate, the former being a complex, polyampholytic protein bearing 18 of the 20 amino acids and the latter a relatively simple polysaccharide, which is a copolymer of β-d-mannuronate and α-l-guluronate, each with a carboxylate group. Alginate is a well-known gel-forming polymer where stacking of the saccharides between chains occurs in conjunction with Ca ions and has an “eggbox” structure.^37^

While there was no gelation in the low concentration alginate solutions (0.001 mg/mL) dialyzed with NaCl and Gd, there is clearly association occurring, and this association is disrupted by Gd. Hence, it seems that the associations occurring under the dialysis conditions here are due to HP effects. As such, the same type of HP potential energy, ⟨*U*_HP_⟩, used for gelatin can be used here, even though the molecular basis of the HP effects may be quite different between amino acids and uronic acid sugars.

Eqs 9a and 9b for gelatin can be expressed for alginate, in the absence of polyampholyte effects, where ⟨*U*_el,A_⟩ ≥ 0, so that ⟨*U*_el,net_⟩ ≥ ⟨*U*_el,E_⟩
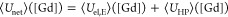
23a
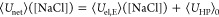
23b

**Figure 10:**
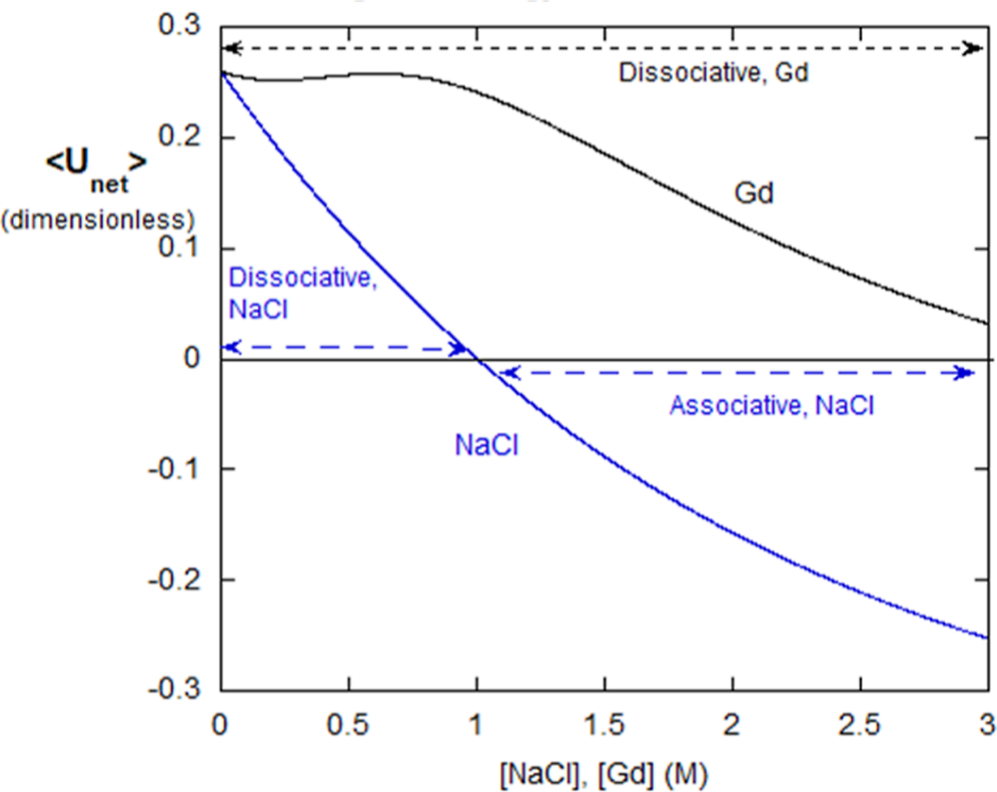
⟨*U*_net_⟩ for alginate in Gd and in NaCl. At very low IS, both Gd and NaCl have negative potentials, leading to interchain associations. Gd, because it suppresses HP effects quickly leads to a positive ⟨*U*_net_⟩ and dissociation of alginate chains. In contrast, *U*_net_ for NaCl remains negative throughout, causing further irreversible aggregation, as seen in Figure 9.

Eqs 6a and 6b can be used directly for ⟨*U*_HP_⟩, with the understanding that the parameters appearing in them will be different between gelatin and alginate. Figure 10 shows the net potentials for Gd and NaCl versus those of [NaCl] and [Gd]. The model captures the fact that ⟨*U*_net_⟩([NaCl]) versus [NaCl] goes from positive (dissociative) to negative (associative) at a certain [NaCl], 1 M here. Because these associations are irreversible, they are “aggregates” in the current terminology.

In contrast ⟨*U*_net_⟩([Gd]) starts positive (dissociative) and remains positive throughout the range of Gd.

The parameters used in Figure 10 were ⟨UHP⟩_0_ = −0.40, β_E_ = 0.5, *U*_el,0_ = 0.66, [Gd]_1/2_ = 0.3 M, γ = 3.0 (1/M), [NaCl], [Gd] = 2.5κ^2^. These parameters are merely illustrative and rationalize the monitored associative and dissociative behavior.

### 3.4 Selection of Proteins

#### 3.4.1 BSA; A, E, and HP Interactions

BSA lots from Sigma gave results that varied with the specific batches. An earlier work on thermally induced aggregation of BSA showed that there was a very wide variation in aggregation rates for nominally identical BSA from Sigma, but from differently dated lots,^38^ so this inconsistency in aggregation behavior among lots has now been observed in both thermal stress and, in this work, during dialysis. At this point, there is no conclusive explanation for the variations.

**Figure 11:**
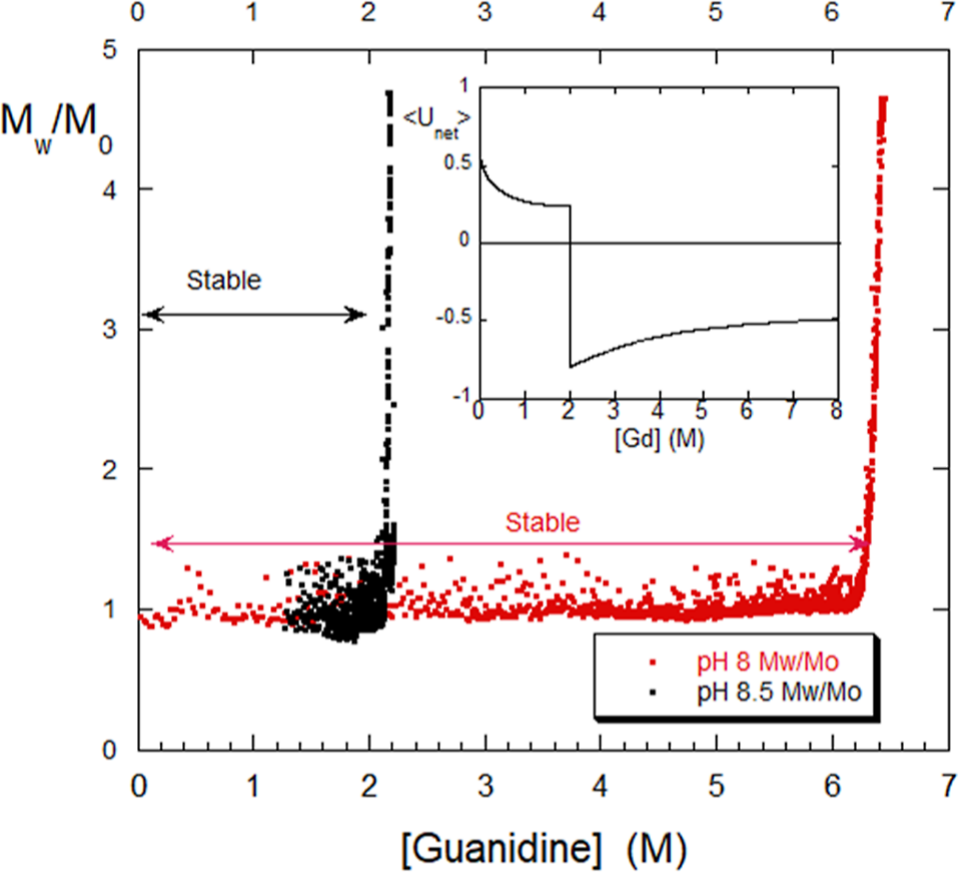
Dialysis of BSA against Gd, showing thresholds for onset of abrupt colloidal aggregation. The process is irreversible in both cases. The inset shows the energy model, where the postulated attractive polyampholyte potential turns on abruptly at the aggregation threshold.

The BSA lots from Sigma were acquired between October 2021 and November 2023. The stated purity was 98% for some lots and 99% for others. 99% pure BSA from June 2023 showed a sharp, reproducible, pH-dependent aggregation threshold versus [Gd], as shown in Figure 11. The 98% pure BSA showed aggregation, stirred and unstirred, but without a sharp aggregation threshold (data not shown). Other lots showed no aggregation, whether stirred or unstirred.

With these comments in mind, Figure 11 shows the results of BSA dialysis (99% pure, 2 mg/mL, Sigma lot from June 2023, unstirred) against 6.5 M Gd, where the BSA is buffered with PBS at pH 8.0 in one dialysis run and at pH 8.5, buffered with Tris in the other. The data are shown in terms of *M*_w_(*t*)/*M*_0_, where *M*_0_ is the initial unaggregated scattering from BSA, around 65,000 g/mol, and *M*_w_(*t*) is the weight-average molar mass of all BSA in the solution, in both native and aggregated forms. The isoelectric point of BSA is well below the lowest pH used (8.0), so it should have a net negative charge at the pH values here.

Remarkably, there is a very sharp threshold in each case, in which colloidal aggregation sets in. The data go vertically off the scale in each case. It is further striking that there is such a wide difference in stability against Gd due to a fairly small difference in pH. In both cases, the aggregation was completely irreversible. These thresholds were reproducible under repeated dialysis experiments with the same June 2023 batch of BSA.

No threshold or large-scale aggregation was found when dialyzing against NaCl. This implies that the aggregation threshold during dialysis against Gd requires HP interactions. Since Gd suppresses HP effects, it would seem that the aggregation is electrostatic in nature and may correspond to association of opposite charges once the BSA denatures due to Gd. When BSA is intact, the net negative charge keeps it from aggregating (the opposite charges on two chains cannot overcome this barrier to interact), but once the structure opens up, there is the possibility of closer opposite charge associations that are suppressed when the BSA is in the globular form.

Following the above model of combined electrostatic and HP potentials, it can be surmised that below the threshold only ⟨*U*_el,E_⟩ and ⟨*U*_HP_⟩ are operative, and the polyampholyte potential ⟨*U*_el,A_⟩ is negligible. Upon unfolding, opposite charges between chains can interact more closely and ⟨*U*_el,A_⟩, which is negative (attractive) abruptly becomes much more important. When this potential “turns on”, ⟨*U*_net_⟩ goes negative and the aggregation ensues. The inset to Figure 11 shows what such a net potential might look like, where the same parameters as shown in Figure 8 are used, except in Figure 11 ⟨*U*_el,A_⟩ is zero until the aggregation threshold at [Gd]_threshold_ = 2 M for pH 8.5, at which point ⟨*U*_el,A_⟩ turns on with the form .

#### 3.4.2 Lysozyme; E and HP Interactions

Lysozyme dialysis against 1 M Gd showed no effect. Measuring possible time-dependence in separate nondialysis experiments showed no time-dependent effects, including up to 4 M Gd. In contrast, NaCl caused time-dependent aggregation of lysozyme after 0.5 M NaCl. Hence, running NaCl dialysis crosses time scales with aggregation that occurs at fixed [NaCl], so the data are not quantitatively meaningful. Under dialysis against 4 M NaCl, the lysozyme showed immediate aggregation, leading to strong particulate formation. Upon reverse dialysis, the time-dependent aggregation continued.

**Figure 12:**
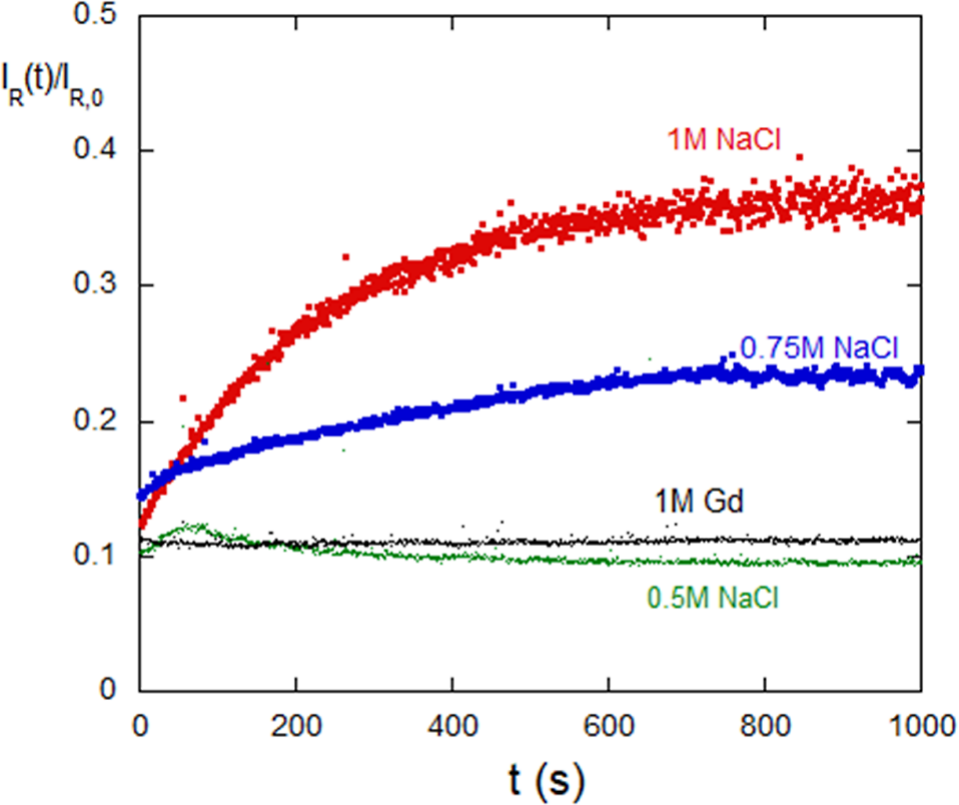
Aggregation of lysozyme above 0.5 M NaCl at fixed [IS] (i.e., nondialysis). Nonaggregation regime starts somewhere between 0.5 and 0.75 M NaCl, continuing to 0 NaCl. Nonaggregation was found over 0–4 M Gd.

Figure 12 shows the time-dependent aggregation of lysozyme for fixed values of [NaCl] above 0.5 M NaCl (no dialysis). It also shows no measurable aggregation at (and below) 0.5 M NaCl. The nonaggregating data for 1 M Gd (no dialysis) are shown in Figure 12, and nonaggregation continued until 4 M Gd.

Following the electrostatic/HP energetic model above, the results are similar to those for alginate, Figure 10, except for several distinguishing features: (i) the strong time dependence of aggregation for lysozyme, (ii) ⟨*U*_net_⟩ for Gd is positive throughout (unlike in Figure 10, there is no low [Gd] negative potential, as seen for alginate), and so no aggregation occurs, and (iii) ⟨*U*_net_⟩ is positive for NaCl up until 0.5 M, after which it becomes negative, leading to aggregation NaCl (whereas in Figure 10 for alginate, ⟨*U*⟩_net_([NaCl] is always negative).

#### 3.4.3 IgG; E and HP Interactions

**Figure 13:**
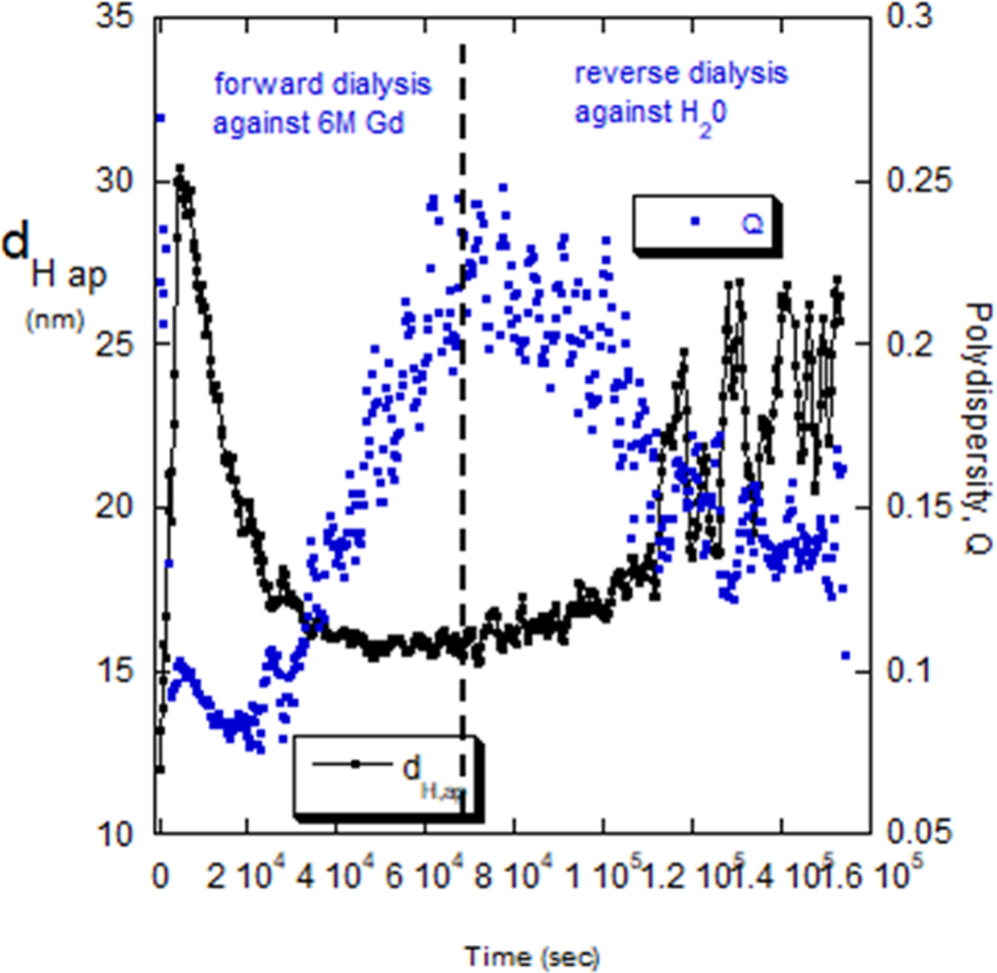
*d*_H,ap_ and *Q* for IgG undergoing forward dialysis against 6 M Gd and reverse dialysis against water.

IgG has a mass around 150,000 g/mol and presents an interesting profile under dialysis against 6 M Gd, as seen in Figure 13. There is an abrupt rise in *d*_H,ap_ at very low [Gd], from 12 to 30 nm, after which *d*_H,ap_ falls monotonically as [Gd] increases, down to 16 nm. Upon reverse dialysis, *d*_H,ap_ increases from 16 nm to about 27 nm; i.e., it approaches the forward dialysis value it had after the initial abrupt increase.

I_R_ almost exactly mirrors the trend of *d*_H,ap_ in the forward and reverse dialysis, so the second *y*-axis in Figure 13 is used instead to show polydispersity, *Q*, from the DLS second cumulant analysis. At a glance, it supports the partial reversibility of the dialysis. Interestingly, *Q* increases as d_H,ap_ decreases.

An interpretation of the data is that the ionic strength provided at low [Gd] is enough to shield net charge between the IgG, and HP effects cause association, perhaps to an oligomeric form, such as a dimer, tetramer, or higher. This oligomeric form may be well-defined because *Q* drops precipitously from 0.27 (medium polydispersity) to <0.10 (low polydispersity) after the abrupt increase in *d*_H,ap_. It is further surmised that as [Gd] increases, the HP effects are interrupted, and the oligomers dissociate, but nonuniformly, so that there is a spread of *d*_H,ap_ among the associations, leading to markedly increasing polydispersity, back to its original value by the end of the forward dialysis.

Upon reverse dialysis against water there is a reassociation which “gathers up” the dissociated fragments and reassembles them toward the well-defined oligomeric form after the initial abrupt increase, while *Q* decreases. Hence, the fragmentation and buildup of oligomers in the forward and reverse dialyses, respectively, are at least partially reversible.

**Figure 14:**
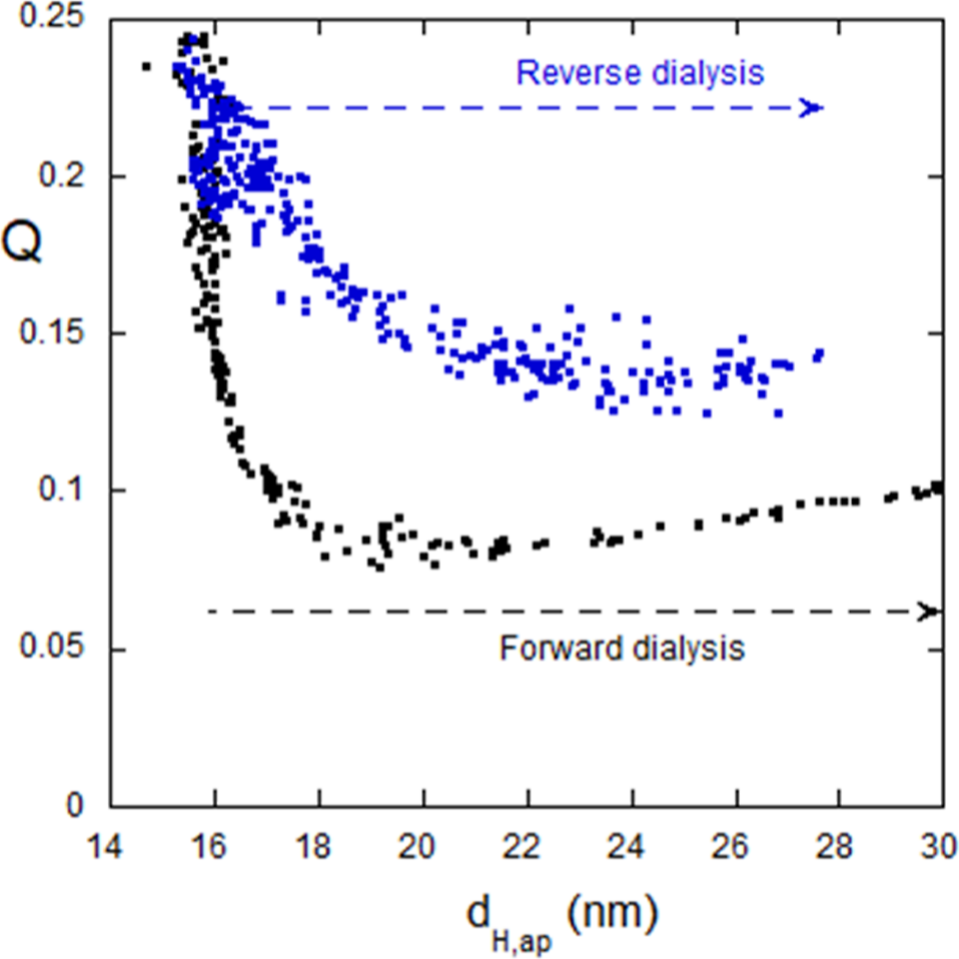
*Q* vs *d*_H,ap_ in forward dialysis of IgG versus 6 M Gd and reverse against water. Remarkably, *Q* increases as *d*_H,ap_ decreases, suggesting the inhomogeneous fragmentation of the oligomer formed at the maximum of *d*_H,ap_ in Figure 13.

This conjectured fragmentation of a “stable” oligomeric form with accompanying increasing polydispersity and its reverse process can be better appreciated in Figure 14. In most aggregation processes, *Q* increases or stays the same with increasing aggregation and *d*_H,ap_; Figure 14 shows a remarkable deviation from this trend.

The forward dialysis profile of IgG versus Gd is qualitatively similar to forward dialysis of alginate versus Gd in Figure 9; an initial rise in scattering intensity followed by a steady decrease. The difference is that in reverse dialysis alginate is not at all reversible, whereas the IgG oligomers are at least partially reversible. The electrostatic and HP potential profiles for IgG may resemble ⟨*U*_net_⟩([Gd]) for alginate dialysis against Gd, as shown in Figure 10, including the initial negative portion. This difference in reversibility between alginate and IgG suggests an ordered, associated structure in the reversible case of IgG, as opposed to a random colloidal aggregate for alginate. This seems plausible because globular proteins are known to functionally associate *in vivo*, whereas alginate, resembling a random coil in space, has no symmetry that might lead to an organized structure.

#### 3.4.4 Proteinase K; E and HP Interactions

**Figure 15:**
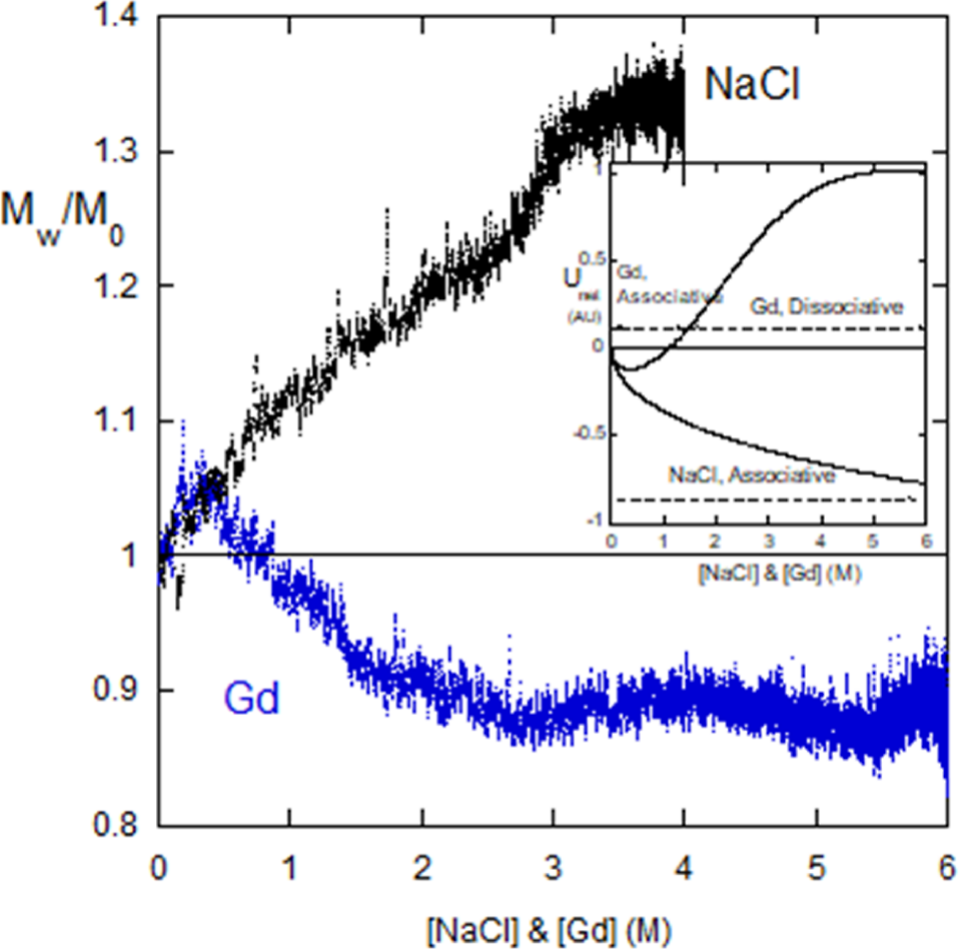
Contrast between NaCl and Gd forward dialysis for proteinase K. The inset shows ⟨*U*_net_⟩ for NaCl and Gd. (v) Casein micelles; E, A, HP, and intraparticle interactions.

Figure 15 shows the contrast of proteinase K behavior under dialysis from pure water against 4 M NaCl and from pure water against 6 M Gd. With NaCl, aggregation sets in immediately and climbs to a maximum. With Gd, there is initial aggregation until about 0.5 M Gd, after which dissociation begins. *M*_w_/*M*_0_ drops below 1.0 at about 0.8 M, suggesting that there were some associations in the initial solution, which after aggregating at low [Gd] then dissociate.

The behavior is qualitatively similar to that of alginate in NaCl and Gd dialysis. This was interpreted with the screened potential of eq 16a and HP potential of eqs 18a,b.

The inset to Figure 15 shows the energy model, which involves the ⟨*U*_HP_⟩ from eq 6a (Gd) and 6b (NaCl), and the repulsive polyelectrolyte potential of eq 5a. The parameters are β = 1, *r*_e_ = 0.18, [Gd]_1/2_ = 2 (M), γ = 1 (1/Μ), *U*_hp,o_ = −2.0, and *U*_el,e,0_ = 2.0. The energy model in Figure 15 inset is qualitatively similar to the alginate potential in Figure 10, including the negative potential at low [Gd] causing associations to form before ⟨*U*_net_⟩ turns positive at around 0.5 M Gd.

**Figure 16:**
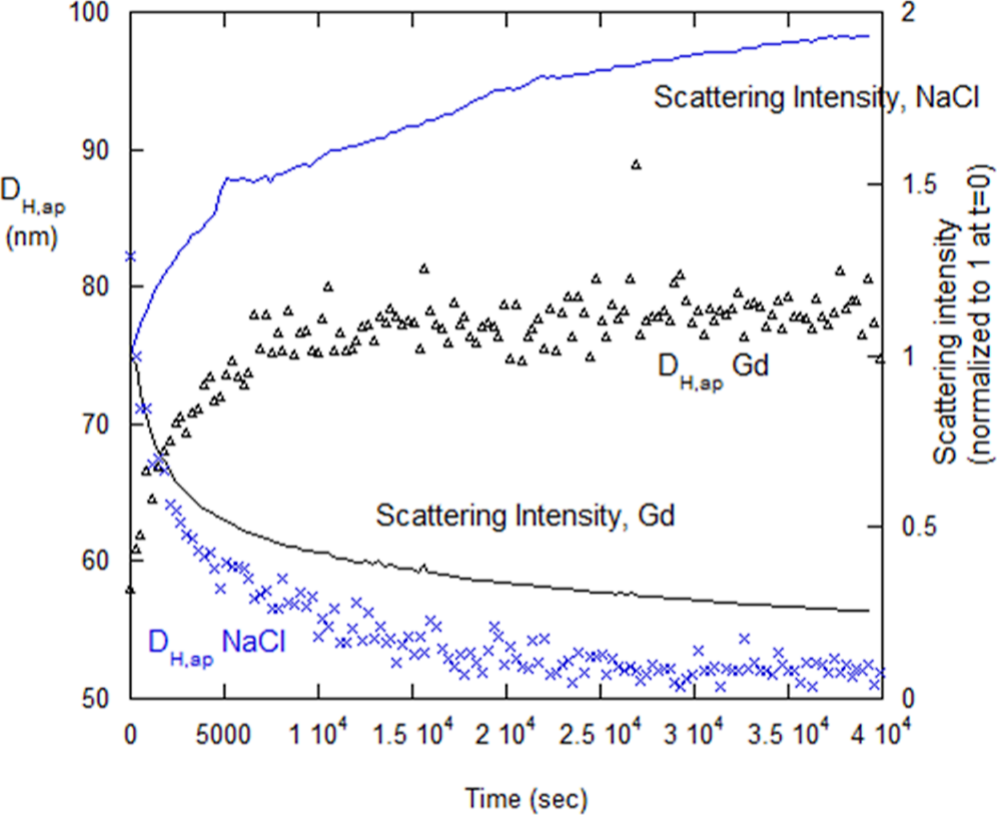
Contrast in dialysis behavior against NaCl and Gd for casein.

Casein presents an unusual dialysis profile, against both NaCl and Gd, Figure 16. As NaCl increases, the scattering intensity increases but *d*_H,ap_ decreases. The Gd case is exactly the opposite; as Gd increases, scattering decreases but *d*_H,ap_ increases. *Q* decreases for NaCl and increases for Gd (data not shown).

It should first be noted that casein micelles are quite complex and are not fully understood. There are several different models for the structure of casein micelles, all of which are plausible, but none of which is definitive.^39−42^ The immediate purpose here is to show the new data resulting from the monitored dialysis as a means of providing complementary information to understand more about the micellar behavior and not to solve the structural problem. The interpretive model is used next to attempt a partial phenomenological conjecture of the data trends.

The phosphoproteins in casein micelles give them a strong negative charge in the aqueous solution used. The micelles are held together by HP and electrostatic interactions. Hence, it can be conjectured that the increase in intensity with increasing [NaCl] is the usual polyelectrolyte effect of charge screening between casein micelles with subsequent lowering of A_2_, such as for PSS in Figures 2 and 3. The fact that *d*_H,ap_ simultaneously decreases suggests that there are attractive intramicellar polyampholyte effects that are suppressed, causing the micelles to partially dissociate into smaller micelles, perhaps subunits, that are still highly charged. If true, this implies that casein micellization is affected by both polyampholyte and HP interactions. The decreasing *Q* with [NaCl] suggests that the smaller micelles have a narrowing size distribution.

While Gd at low concentration has a simple polyelectrolyte effect early in the Gd dialysis of alginate, an initial rise in scattering, before its HP interactions begin to dominate and intensity falls, in the Gd dialysis of casein there is an immediate decrease in scattering intensity at low [Gd], which continues to decrease as [Gd] increases. Hence, the ability of Gd to disturb the micelles seems to begin immediately as Gd dialysis begins, and this thoroughly dominates any polyelectrolyte effect. A conjecture is that Gd begins to immediately weaken the HP interactions within the micelles, allowing water to penetrate into the interior, swelling the micelles, thus increasing *d*_H,ap_ and/or changing the morphology, e.g., from spheres to rods or fibers.

The interpretive model as it stands only considers interparticle E, A, and HP interactions and does not take account of intraparticle E, A, and HP interactions. The extension of the model to include both inter- and intraparticle effects is left to a next phase of research.

## 4 Conclusions

Monitoring light scattering behavior of macromolecules undergoing membrane dialysis against a salt (NaCl) and a denaturant (Guanidine-HCl) revealed an intricate interplay of potential energies: positive (repulsive) polyelectrolyte (E), negative (attractive) polyampholyte (A), and associative H-bond/HP potential energies. An interpretive, dimensionless model based on these potential energies rationalizes the associative, aggregative, and dissociative behavior of the protein and polysaccharide biomacromolecules monitored. No attempt was made here, however, to define the absolute strengths of the potentials and find fits to the data. Rather, dimensionless analysis was used to understand the results, and an analysis of the absolute values of the potentials related to the macromolecular nanostructure is left to further work.

Table 1 summarizes the observations and behavior for the various macromolecules.

PSS was the only nonbiological macromolecule investigated. Because only the repulsive polyelectrolyte potential ⟨*U*_el,E_⟩ was operative over the range of ionic strength used, the dialysis was fully reversible. Detailed information was obtained on the ionic strength dependence of the second and third virial coefficients, *A*_2_ and *A*_3_, as well as the hydrodynamic interaction term *k*_D_ in the diffusion coefficient expansion of eq 18. A linear relationship was found between *A*_2_ and the hydrodynamic interaction parameter *k*_D_.

The dialysis monitoring method can be used in many contexts. Effects on macromolecules and colloids can be found for different types of small molecules, such as electrolytes of varying species, valences, and symmetry, denaturants, chelating agents, surfactants, and excipients in general. Effects of the solution tonicity on cells and organelles can also be monitored. Because the dialysis apparatus fits into any standard 1 cm cuvette, it can be used with any instrument that accepts such cuvettes, including UV/visible, fluorescence, and circular dichroism spectrometers.

It is hoped that this approach will both offer new insights into the physics of macromolecules and aid in the formulation and stability of biologic drugs. In the latter case, regimes of stability versus concentration of different electrolytes and agents can be determined as the dialysis process sweeps across a continuous range of concentrations.
